# *PKM2* is a potential prognostic biomarker and related to immune infiltration in lung cancer

**DOI:** 10.1038/s41598-023-49558-4

**Published:** 2023-12-14

**Authors:** Lan Yin, Jiaying Shi, Jingfei Zhang, Xinyu Lin, Wenhao Jiang, Yingchuan Zhu, Yue Song, Yilu Lu, Yongxin Ma

**Affiliations:** Department of Medical Genetics, West China Hospital, Sichuan University, Chengdu, China

**Keywords:** Cancer, Immunology, Biomarkers, Oncology

## Abstract

Pyruvate kinase M2 (*PKM2*), a subtype of pyruvate kinase, plays a crucial role as a key enzyme in the final step of glycolysis. It is involved in regulating the tumor microenvironment and accelerating tumor progression. However, the relationship between *PKM2* expression and the prognosis and immune infiltration remains unclear in lung cancer. In this study, we analyzed *PKM2* expression in pan-cancer, and investigated its association with prognosis and immune cell infiltration of lung cancer by using multiple online databases, including Gent2, Tumor Immune Estimation Resource (TIMER), Gene Expression Profiling Interactive Analysis (GEPIA), PrognoScan, Kaplan–Meier plotter, and The Human Protein Atlas (HPA). The results showed that *PKM2* expression is elevated in tumor tissues compared with the adjacent normal tissues of most cancers, including lung cancer. Prognostic analysis indicated that high expression of *PKM2* was associated with poorer prognosis in overall lung cancer patients, especially in lung adenocarcinoma (LUAD). Notably, *PKM2* exhibited a strong correlation with B cells and CD4+ T cells in LUAD; and with B cells, CD8+ T cells, CD4+ cells, and macrophages in lung squamous cell carcinoma (LUSC). Furthermore, *PKM2* expression displayed a significant negative correlation with the expression of immune cell markers in both LUAD and LUSC. These findings suggested that *PKM2* could serve as a promising prognostic biomarker for lung cancer and provided insights into its essential role in modulating the immune cell infiltration.

## Introduction

According to the World Health Organization's Global Cancer Observatory (https://gco.iarc.fr/), lung cancer has the highest mortality rate in the world, ranking third in terms of incidence. Lung cancer is classified into two main types: small cell lung cancer (SCLC) and non-small cell lung cancer (NSCLC), with approximately 85% of patients diagnosed with NSCLC^1,2^. Lung adenocarcinoma (LUAD) and lung squamous cell carcinoma (LUSC) are the most common subtypes of NSCLC^3^. The occurrence and progression of NSCLC involve a dynamic and intricate process closely linked to the tumor microenvironment, where immune cells can significantly impact cancer cell growth and development^4^. Therefore, there is an urgent need to identify a new therapeutic target associated with the prognosis and immune infiltration of lung cancer.

*PKM2*, a subtype of pyruvate kinase, is highly expressed in proliferating and tumor cells^5^. *PKM2* predominantly exists in the form of monomer and dimer, functioning as a crucial rate-limiting enzyme in glycolysis and a significant regulator of tumor metabolism^6,7^. Its enzyme activity is intricately regulated, enabling cells to adapt to diverse physiological states^8^. Recent research has increasingly focused on the role of *PKM2* as a vital regulator of cellular pathological and physiological activities in autoimmune responses and inflammatory processes^9^. Studies have shown that *PKM2* could modulate the formation of T cell subpopulations and affect T cell metabolism, while also exerting regulatory effects on B cells, dendritic cells, and tumor-associated macrophages^10^. Notably, dimerized *PKM2* has been reported to bind to the promoter of Programmed Cell Death-Ligand 1 (PD-L1), resulting in a significant increase of PD-L1 expression, thereby facilitating immune evasion by cancer cells^11^. Wang et al. have proposed that exosomal *PKM2* may serve as a promising biomarker and therapeutic target for cisplatin resistance in NSCLC^12^. However, the precise function and mechanism of *PKM2* in the progression of lung cancer remain elusive.

In this study, we conducted a comprehensive analysis of *PKM2* expression and its correlation with prognosis in various tumor types. The mRNA expression levels of *PKM2* in tumors and adjacent normal tissues of the LUAD and LUSC were compared using the Kaplan–Meier plotter. Additionally, the protein expression levels of PKM2 were examined using The Human Protein Atlas (HPA) database. Furthermore, we systematically investigated the relationship between *PKM2* expression and immune infiltration in these two subtypes of NSCLC. Our findings revealed that *PKM2* is significantly upregulated in lung cancer and can serve as a valuable biomarker for prognostic prediction. Moreover, our results suggested that *PKM2* may modulate tumor immunity by regulating immune cell infiltration in NSCLC.

## Results

### The mRNA expression levels of *PKM2* in pan-cancer

**Figure 1:**
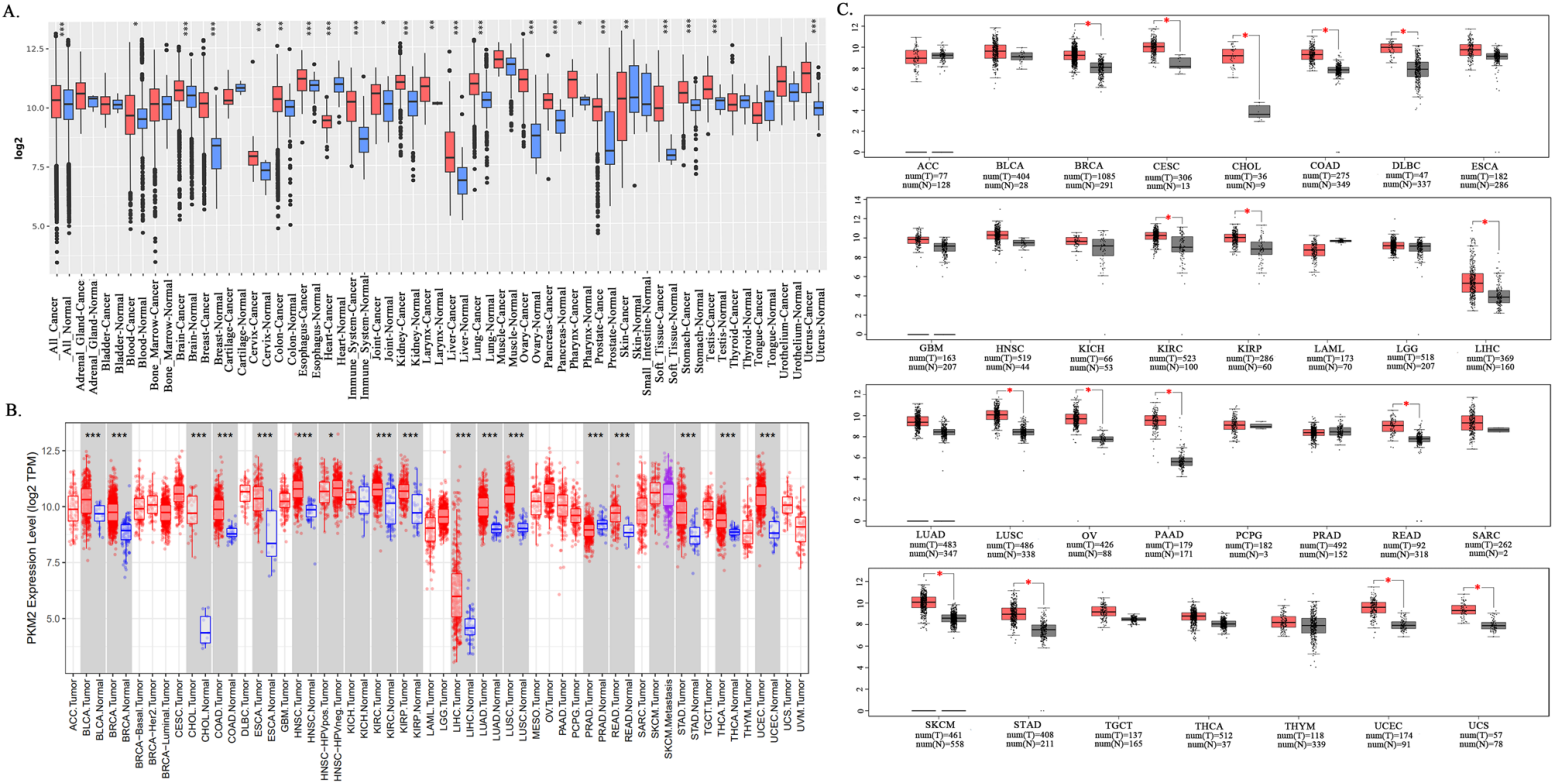
Analysis of *PKM2* expression levels in pan-cancer. (**A**) Comparison of the expression level of *PKM2* in tumors and adjacent normal tissues in pan-cancer using the Gent2 database. (**B**) The expression level of *PKM2* in different cancers and adjacent normal tissues in the TIMER database. (**C**) The expression levels of *PKM2* in different cancers and adjacent normal tissues were compared using the GEPIA database. (*p < 0.05, **p < 0.01, ***p < 0.001).

Figure 1A illustrates the widespread upregulation of *PKM2* in tumors compared with adjacent normal tissues across various cancer types (all *p* < 0.05) in the Gent2 database (GPL96 platform). However, *PKM2* was downregulated in heart and skin cancer tissues (*p* < 0.05). Next, we used TIMER to verify the differential expression of *PKM2* in pan-cancer. With the exception of prostate adenocarcinoma (PRAD), where *PKM2* expression was higher in adjacent normal tissues than in tumor tissues, other cancers exhibited overexpression of *PKM2* in tumor tissues (Fig. 1B). In addition, we investigated the mRNA expression levels of *PKM2* in tumors and adjacent normal tissues from the TCGA and GTEx datasets using GEPIA database (Fig. 1C). The results revealed higher *PKM2* mRNA levels in breast invasive carcinoma (BRCA), cervical squamous cell carcinoma and endocervical adenocarcinoma (CESC), cholangio carcinoma (CHOL), colon adenocarcinoma (COAD), lymphoid neoplasm diffuse large B-cell lymphoma (DLBC), kidney renal clear cell carcinoma (KIRC), kidney renal papillary cell carcinoma (KIRP), liver hepatocellular carcinoma (LIHC), LUSC, ovarian serous cystadenocarcinoma (OV), pancreatic adenocarcinoma (PAAD), rectum adenocarcinoma (READ), skin cutaneous melanoma (SKCM), stomach adenocarcinoma (STAD), uterine corpus endometrial carcinoma (UCEC) and uterine carcinosarcoma (UCS) than in adjacent normal tissues (all *p* < 0.05). However, no statistical differences in the expression of *PKM2* were observed between tumor and adjacent normal tissues in other tumors.

### Analysis of the prognostic value of *PKM2* in human cancer

**Figure 2:**
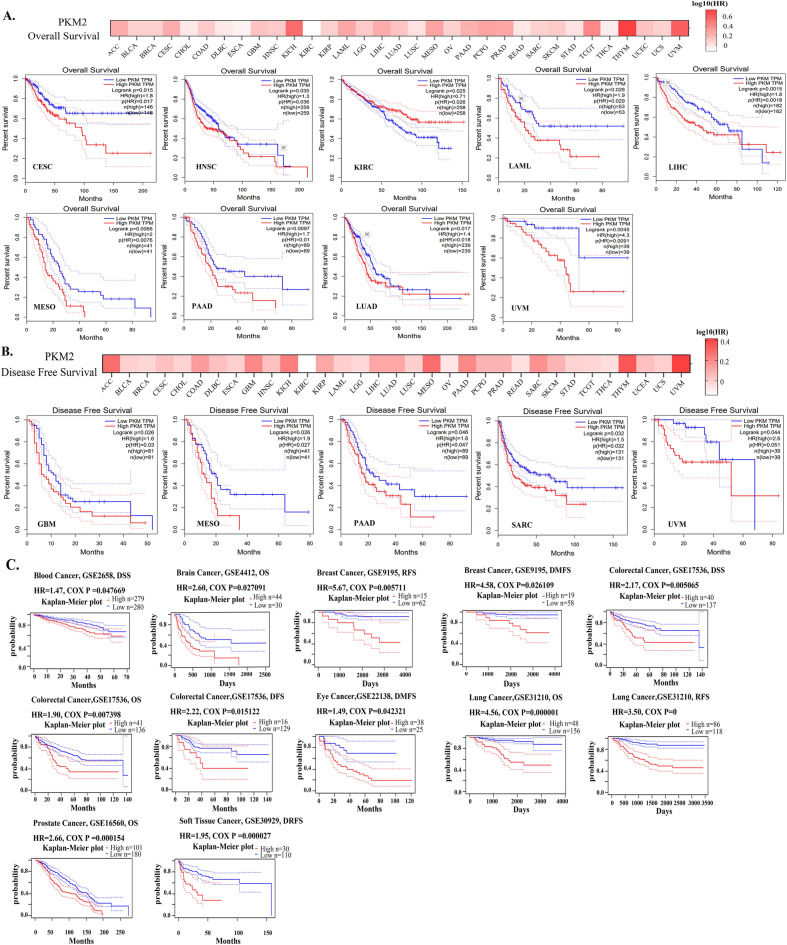
Analysis of the prognostic significance of *PKM2* in different types of cancers. The relationship between *PKM2* expression and overall survival (**A**) and disease-free survival (**B**) in different cancers was analyzed by GEPIA database. (**C**) The prognostic value of *PKM2* in different cancers was analyzed using the PrognoScan database.

The prognostic value of *PKM2* in cancers was evaluated using GEPIA and PrognoScan databases. In the GEPIA database, high expression of *PKM2* was associated with worse OS in CESC, head and neck squamous cell carcinoma (HNSC), acute myeloid leukemia (LAML), LIHC, LUAD, mesothelioma (MESO), PAAD, and uveal melanoma (UVM), but was associated with a better OS in KIRC (Fig. 2A; Table S1). In addition, high expression of *PKM2* was negatively associated with disease-free survival (DFS) in glioblastoma multiforme (GBM), MESO, PAAD, and UVM (Fig. 2B). Similarly, *PKM2* expression was shown to be negatively correlated with the prognosis of 8 out of 12 cancers (cox *p* < 0.05), including blood, brain, breast, colorectal, eye, lung, prostate, and soft-tissue cancers (Fig. 2C).

### Expression analysis of *PKM2* in lung cancer

**Figure 3:**
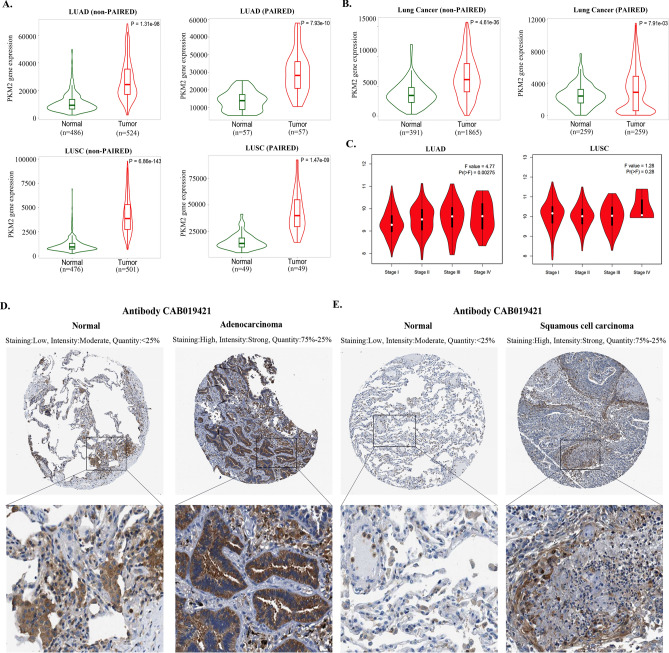
Analysis of the mRNA and protein expression levels of PKM2 in tumor and adjacent normal tissues of lung cancer. RNA-seq (**A**) and gene chip (**B**) data from the KM plotter database were used to compare the expression levels of *PKM2* in tumor and adjacent normal tissues. (**C**) Correlation between *PKM2* expression and tumor stage in LUAD and LUSC (GEPIA database). (**D**) *PKM2* protein expression levels in tumors and adjacent normal tissues of LUAD and LUSC patients were detected by immunohistochemistry in the HPA database.

Given the exceptionally high global mortality rate and the third-highest incidence rate of lung cancer, we focused towards exploring the potential role of *PKM2* as a biomarker in lung cancer in our subsequent research. The differential expression of *PKM2* in lung cancer was verified using the KM plotter database. Both the RNA-seq based data (Fig. 3A) and the gene chip-based data (Fig. 3B) demonstrated that *PKM2* mRNA expression levels were higher in tumor tissues compared to adjacent normal tissues, regardless of whether non-paired or paired samples were used as controls. Meanwhile, we analyzed the expression of *PKM2* at different stages of LUAD and LUSC using the GEPIA database. The results indicated significant variations in *PKM2* expression levels across different stages in LUAD (Pr(> F) < 0.05; Fig. 3C). Subsequently, we confirmed the protein expression of PKM2 in LUAD and LUSC by immunohistochemistry analysis in the HPA database. The findings revealed elevated protein levels of PKM2 in both LUAD and LUSC tumor tissues compared to paired adjacent normal tissues (Fig. 3D,E).

### Association of *PKM2* expression with prognosis and clinical characteristics of lung cancer patients

**Figure 4:**
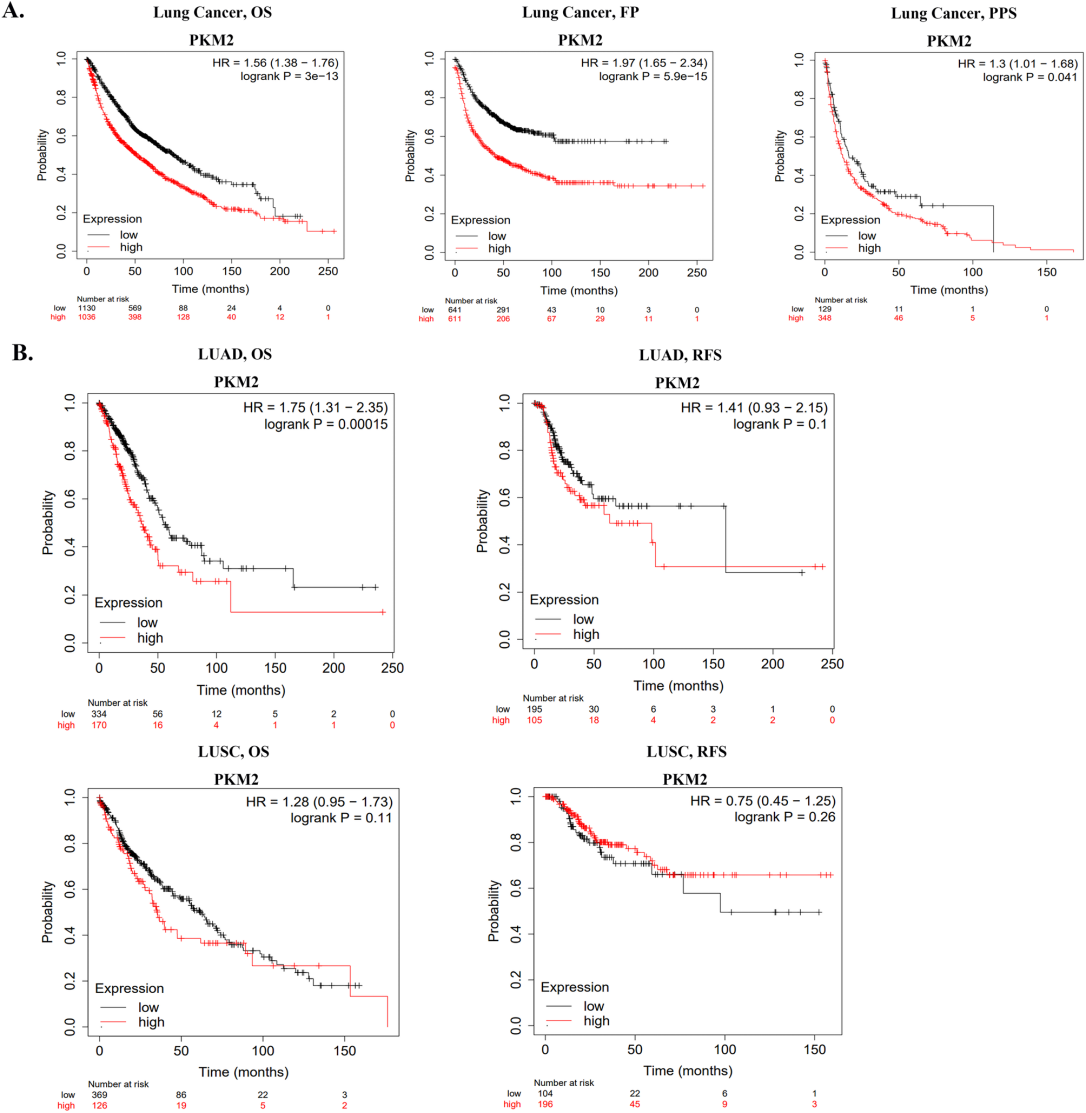
Survival curves for *PKM2* in lung cancer using the Kaplan–Meier plotter database. (**A**) OS, FP, and PPS of lung cancer based on the gene chip data. High expression of *PKM2* was correlated with poor OS, FP and PPS. (**B**) OS and RFS of LUAD and LUSC based on the RNA-seq data.

The effect of *PKM2* expression on prognosis of lung cancer patients was further determined using the KM plotter database. The results of gene chip-based data showed that high expression of *PKM2* was associated with poor OS (HR = 1.56, *p* = 3e−13), the first progression (FP, HR = 1.97, *p* = 5.9e−15), and post progression survival (PPS, HR = 1.3, *p* = 0.041) in lung cancer patients (Fig. 4A). However, the analysis of RNA-seq based data showed that high expression of *PKM2* only correlated with poor OS (HR = 1.75, *p* = 0.00015) in LUAD, and it was not associated with relapse-free survival (RFS) in LUAD or OS and RFS in LUSC (Fig. 4B). These findings suggested that *PKM2* expression has prognostic value for overall lung cancer, while its association with prognosis is specific to LUAD among the two most common subtypes of NSCLC.

Meanwhile, we examined the relationship between *PKM2* expression and various clinical characteristics of lung cancer using the KM plotter database. As demonstrated in Table 1, high expression of *PKM2* had a detrimental effect on OS, FP, and PPS in specific subgroups, including females (OS: HR = 1.69, *p* = 2.4e−06; FP: HR = 2.5, *p* = 3.8e−11; PPS: HR = 1.57 *p* = 0.017), LUAD patients (OS: HR = 2.64, *p* = 1.1e−15; FP: HR = 2.36, *p* < 1E−16; PPS: HR = 1.41, *p* = 0.03), stage 1 patients (OS: HR = 2.38, *p* = 5.9e−12; FP: HR = 2.18, *p* = 0.00042; PPS: HR = 2.52, *p* = 0.0065), AJCC_N1 (OS: HR = 1.54,*p* = 0.01; FP: HR = 2.62, *p* = 1e−05; PPS: HR = 1.72, *p* = 0.016), non-smokers (OS: HR = 4.56, *p* = 3.1e−06; FP: HR = 3.72, *p* = 2.6e−07; PPS: HR = 2.31, *p* = 0.0081), and patients with negative surgical margins (OS: HR = 3.03, *p* = 2.1e−15; FP: HR = 4.8, *p* = 2.2e−16; PPS: HR = 1.51, *p* = 0.0057). In addition, high *PKM2* expression correlated with poor OS and PFS in males (OS: HR = 1.69, *p* = 6.4e−08; FP: HR = 1.75, *p* = 3.2e−07), AJCC (American joint committee on cancer)_T1 patients (OS: HR = 2.03, *p* = 1.8e−05; FP: HR = 3.08, *p* = 4.8e−07), AJCC_T2 patients (OS: H R = 1.31, *p* = 0.025; FP: HR = 1.46, *p* = 0.0054), AJCC_N0 patients (OS: HR = 1.47, *p* = 0.00095; FP: HR = 1.53, *p* = 0.0022), and patients with a history of smoking (OS: HR = 1.72, *p* = 4.6e−07; FP: HR = 1.97, *p* = 4.4e−08). These findings highlight the prognostic significance of PKM2 expression in different clinical characteristics of lung cancer patients, particularly in localized and regional early and mid-stage cancers.

**Table 1:** Analysis of the correlation between PKM2 mRNA expression and prognosis of different clinicopathologic factors in lung cancer by Kaplan–Meier plotter.

Clinicopathological factors	Overall survival			First progression			Post progression survival		
	N	Hazard ratio	*p* value	N	Hazard ratio	*p* value	N	Hazard ratio	*p* value
Gender									
Female	808	1.69 (1.35–2.1)	**2.4e−06**	500	2.5 (1.89–3.31)	**3.8e−11**	193	1.57 (1.08–2.29)	**0.017**
Male	1247	1.69 (1.4–2.06)	**6.4e−08**	752	1.75 (1.41–2.18)	**3.2e−07**	284	1.28 (0.92–1.78)	0.15
Histology									
Adenocarcinoma	1161	2.64 (2.06–3.38)	**1.1e−15**	906	2.36 (1.93–2.89)	**< 1E−16**	376	1.41 (1.03–1.92)	**0.03**
Squamous cell carcinoma	780	1.36 (1.12–1.65)	**0.0021**	220	1.47 (0.97–2.22)	0.067	51	1.32 (0.74–2.37)	0.34
Stage									
1	656	2.38 (1.85–3.07)	**5.9e−12**	325	2.18 (1.4–3.4)	**0.00042**	78	2.52 (1.27–5.02)	**0.0065**
2	392	2.08 (1.5 -2.88)	**6.3e−06**	130	1.23 (0.73–2.06)	0.44	58	2.16 (1.12–4.15)	**0.019**
3	108	1.26 (0.76–2.08)	0.37	19	–	–	10	–	–
4	4	–	–	0	–	–	0	–	–
AJCC_T									
T1	429	2.03 (1.46–2.83)	**1.8e−05**	338	3.08 (1.94–4.89)	**4.8e−07**	111	0.66 (0.42–1.03)	0.068
T2	578	1.31 (1.03–1.67)	**0.025**	415	1.46 (1.12–1.92)	**0.0054**	221	1.32 (0.95–1.83)	0.095
T3	82	1.57 (0.93–2.63)	0.087	48	2.08 (1.01–4.3)	**0.044**	35	0.85 (0.41–1.75)	0.66
T4	43	0.6 (0.31–1.17)	0.13	25	0.36 (0.1–1.22)	0.087	17	-	-
AJCC_N									
N0	768	1.47 (1.17–1.86)	**0.00095**	562	1.53 (1.16–2.02)	**0.0022**	218	0.87 (0.63–1.21)	0.41
N1	253	1.54 (1.11–2.15)	**0.01**	181	2.62 (1.68–4.09)	**1e−05**	105	1.72 (1.1–2.7)	**0.016**
N2	104	1.12 (0.74–1.7)	0.58	76	0.7 (0.39–1.26)	0.23	55	0.6 (0.34–1.06)	0.078
AJCC_M									
M0	682	1.45 (1.18–1.79)	**0.00038**	464	1.26 (0.93–1.71)	0.13	159	1.16 (0.82–1.64)	0.41
M1	10	–	–	6	–	–	4	–	–
Grade									
I	188	1.34 (0.89–2.02)	0.17	136	0.7 (0.43–1.14)	0.15	88	1.93 (1.2–3.1)	**0.0058**
II	302	1.86 (1.31–2.63)	**0.00043**	166	1.45 (0.97–2.17)	0.07	106	1.4 (0.89–2.18)	0.14
III	75	3.11 (1.58–6.14	**0.00057**	52	0.54 (0.22–1.3)	0.16	28	0.25 (0.07–0.89)	**0.021**
Smoking history									
Yes	833	1.72 (1.39–2.12)	**4.6e−07**	584	1.97 (1.54–2.52)	**4.4e−08**	266	1.28 (0.94–1.75)	0.12
No	204	4.56 (2.27–9.16)	**3.1e−06**	190	3.72 (2.18–6.36)	**2.6e−07**	70	2.31 (1.22–4.35	**0.0081**
Chemotherapy									
Yes	173	1.97 (1.29–2.99)	**0.0013**	122	1.67 (1.04–2.69)	**0.032**	88	2.06 (1.25–3.37)	**0.0036**
No	400	1.63 (1.22–2.19)	**0.00095**	285	1.29 (0.87–1.92)	0.2	153	1.43 (0.92–2.23)	0.11
Radiotherapy									
Yes	65	2.74 (1.51–4.99)	**6e−04**	62	1.51 (0.81–2.82)	0.19	54	2.02 (1.08–3.77)	**0.025**
No	363	1.24 (0.92–1.67)	0.16	289	0.97 (0.7–1.35)	0.87	163	1.12 (0.78–1.6	0.53
Surgery success									
Only surgical margins negative	704	3.03 (2.27–4.04)	**2.1e−15**	558	4.8 (3.18–7.26)	**2.2e−16**	276	1.51 (1.13–2.04)	**0.0057**

Bold values indicate *p* < 0.05.

### Correlation of *PKM2* expression with the level of infiltrating immune cells in LUAD and LUSC

**Figure 5:**
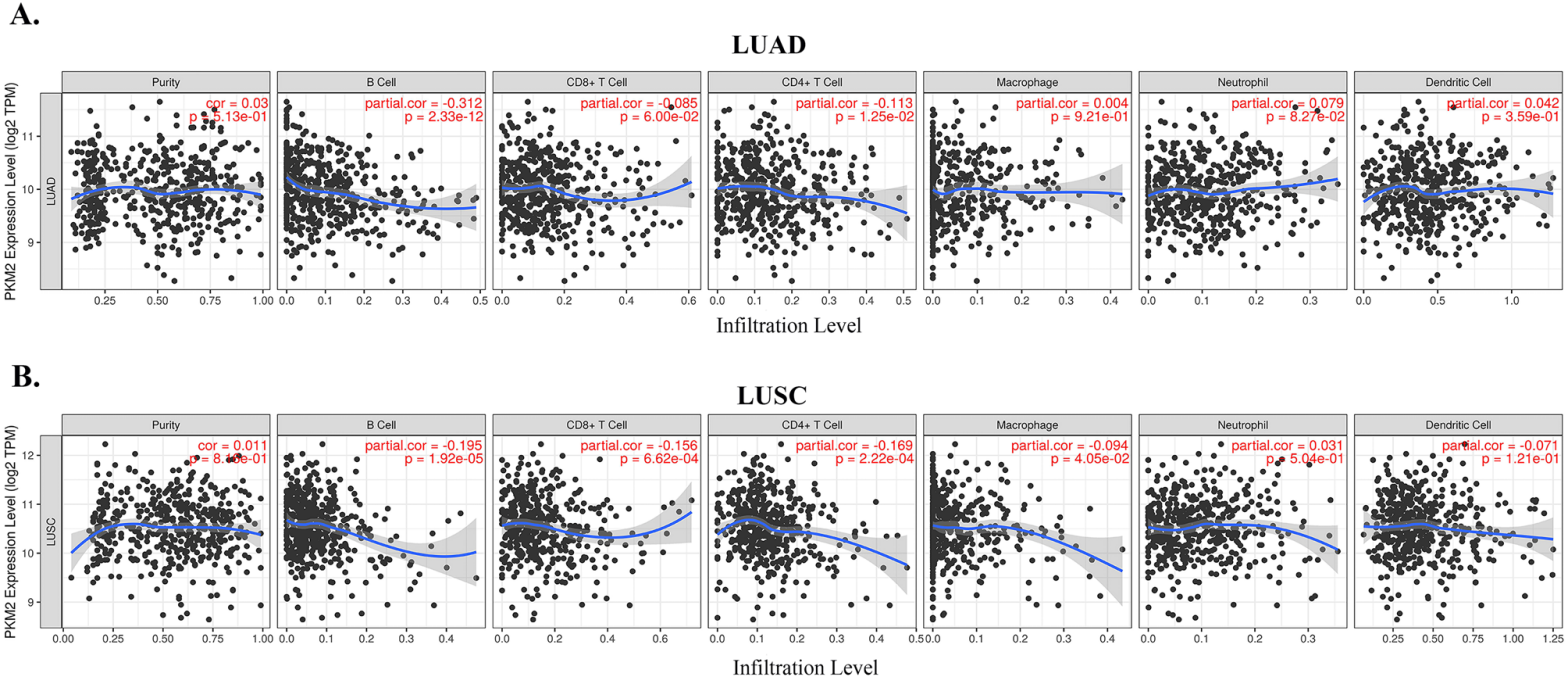
Correlation analysis of *PKM2* expression and infiltration levels of immune cells in LUAD and LUSC tissues using the TIMER database. (**A**) In LUAD, *PKM2* was positively correlated with tumor purity and negatively correlated with the infiltration levels of B-cells and CD4+ T cells. (**B**) *PKM2* expression in LUSC was positively correlated with tumor purity and negatively correlated with infiltration levels of B cells, CD8+ T cells, CD4+ T cells, and macrophages.

Subsequently, we investigated the association between *PKM2* expression and immune cell infiltration in LUAD and LUSC using the TIMER database. As displayed in Fig. 5A and B, the *PKM2* expression levels were positively correlated with the tumor purity (the proportion of cancer cells in a sample) in LUAD and LUSC. The expression level of *PKM2* was significantly negatively correlated with the infiltration levels of B cells (partial. cor = − 0.312, *p* = 2.33e−12) and CD4+ T cells (partial. cor = − 0.113, *p* = 1.25e−02) in LUAD. Similarly, in LUSC, PKM2 expression exhibited a significant negative correlation with the infiltration levels of B cells (partial. cor = − 0.195, *p* = 1.92e−05), CD8+ T cells (partial. cor = − 0.156, *p* = 6.62e−04), CD4+ T cells (partial. cor = − 0.169, *p* = 2.22e−04), and macrophages (partial. cor = − 0.094, *p* = 4.05e−02). These findings highlight the regulatory role of *PKM2* expression in modulating the infiltration of immune cells in LUAD and LUSC.

### Analysis of the correlation between *PKM2* mRNA levels and markers of different immune cell subsets

**Figure 6:**
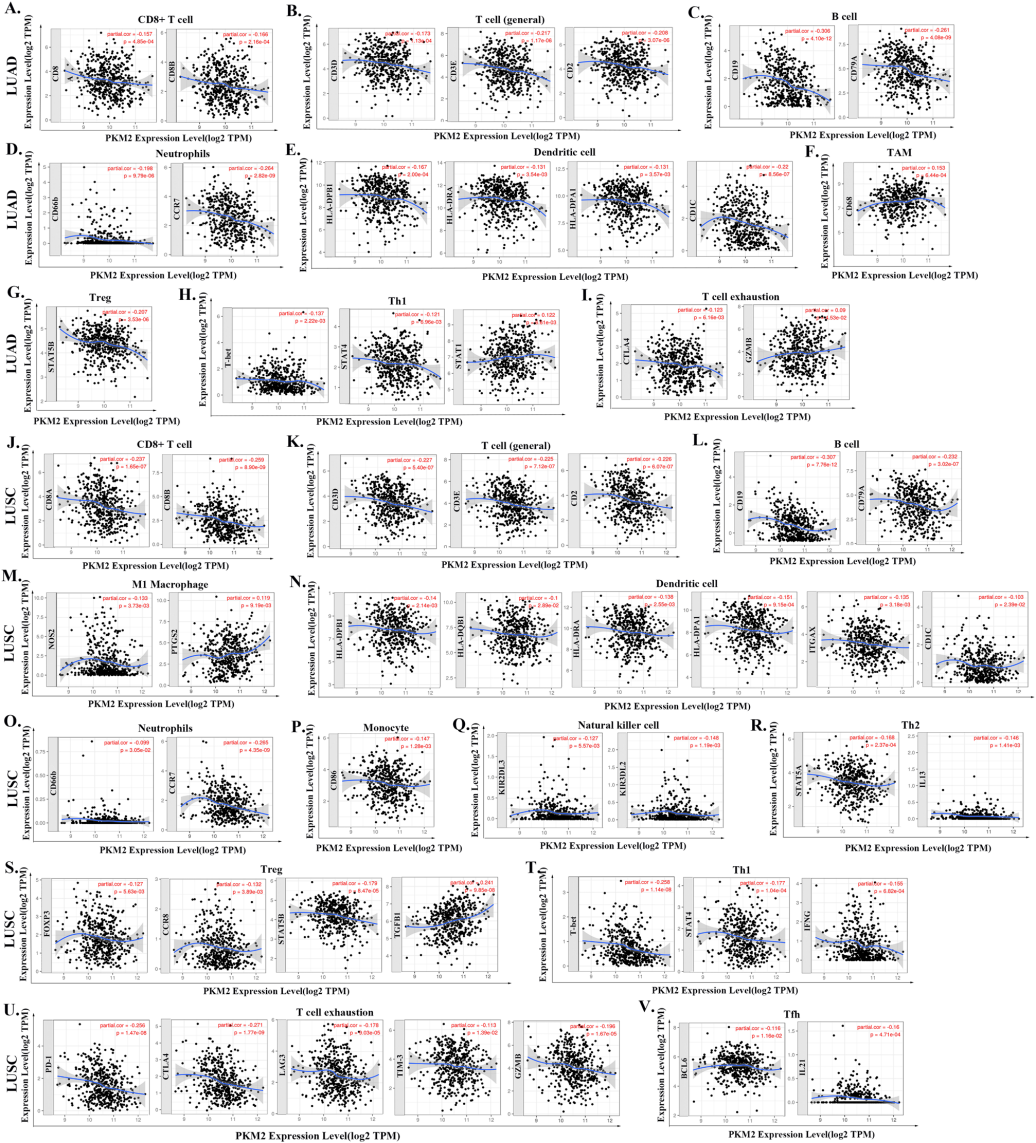
The correlation between *PKM2* expression and levels of infiltrating immune cell markers in LUAD (**A**–**I**) and LUSC (**J**–**V**) was analyzed using the TIMER database. In LUAD, (**A**–**I**) show the correlation between *PKM2* expression and the marker genes in LUAD of CD8+ T cell (CD8A and CD8B) (**A**), T cell (general) (CD3D, CD3E and CD2) (**B**), B cell (CD19 and CD79A) (**C**), Neutrophils (CD66b and CCR7) (**D**), DCs (HLA-DPB1, HLA-DRA, HLA-DPA1, and CD1C) (**E**), TAM (CD68) (**F**), Treg (STAT5B) (**G**), Th1 (T-bet, STAT4, and STAT1) (**H**), and T cell exhaustion (CTLA4 and GZMB) (**I**). In LUSC, (**J**–**V**) show the correlations between CCL14 expression and the marker genes of CD8+ T cell (CD8A and CD8B) (**J**), T cell (general) (CD3D, CD3E, and CD2) (**K**), B cell (CD19 and CD79A) (**L**), M1 Macrophage (NOS2 and PTGS2) (**M**), DCs (HLA-DPB1, HLA-DRA, HLA-DPA1, ITGAX, and CD1C) (**N**), Neutrophils (CD66b and CCR7) (**O**), Monocyte (CD86) (**P**), NK cell (KIR2DL3 and KIR3DL2) (**Q**), Th2 (STAT5A and IL13) (**R**), Treg (FOXP3, CCR8, STAT5B, and TGFβ) (**S**), Th1 (T-bet, STAT4, and IFN-γ) (**T**), T cell exhaustion (PD-1,CTLA4, LAG3, TIM-3, and GZMB) (**U**), and Tfh (BCL6 and IL21) (**V**).

Then, we explored the correlation between *PKM2* mRNA levels and markers of different subsets of immune cells in LUAD and LUSC using TIMER and GEPIA databases^13^. Using TIMER database, purity adjustments were made for correlation analyses given that tumor purity of clinical samples would affect the analysis of immune infiltration (Table 2). In LUAD, *PKM2* expression was negatively correlated with the expression of specific immune cell markers (Fig. 6A–E), including markers of CD8+ T cells, T cells (general), B cells, neutrophils, and DCs. Moreover, *PKM2* expression has a significantly negative correlation with the expression of marker genes of different T cells subsets in LUAD (Fig. 6G–I), such as markers of Treg, Th1 and exhausted T cells. However, a significant positive correlation between *PKM2* and the markers of TAM was observed (Fig. 6F). In LUSC, *PKM2* expression was negatively correlated with the expression of markers of CD8+ T cells, T cells (general), B cells, M1 macrophage, DCs, neutrophils, monocyte, and NK cells (Fig. 6J–Q). Similarly, a negative correlation was observed between *PKM2* expression and different subsets of T cells, including Th2, Treg, Th1, T cell exhaustion, and Tfh (Fig. 6R–V). However, *PKM2* expression had a significant positive correlation with PTGS2 (r = 0.119, *p* = 0.009), a marker of M1 Macrophage, STAT3 (r = 0.114, *p* = 0.013), a marker of Th17, and TGFβ (r = 0.241, *p* = 9.85e−08), a marker of Treg (Table 2). Table 3 shows the correlation analysis between *PKM2* mRNA levels and marker genes of different immune cells in GEPIA database. Collectively, *PKM2* expression was significantly correlated with the expression of marker genes from tumor-infiltrating CD8+ T cells, T cells (general), B cells, Neutrophils, DCs, Th1, Th2, Th17, Tfh, Treg, and depleted T cells both in LUAD and LUSC. Our findings implied a critical role of *PKM2* in tumor immune infiltration in LUAD and LUSC.

**Table 2:** Correlation analysis of PKM2 with immune cell-related genes and markers.

Description	Gene markers	LUAD				LUSC			
		None		Purity		None		Purity	
		Core	*p*	Core	*p*	Core	*p*	Core	*p*
CD8+ T cell	CD8A	− 0.150	**	− 0.157	***	− 0.224	***	− 0.237	***
	CD8B	− 0.166	***	− 0.166	***	− 0.259	***	− 0.259	***
T cell (general)	CD3D	− 0.157	***	− 0.173	***	− 0.208	***	− 0.227	***
	CD3E	− 0.191	***	− 0.217	***	− 0.204	***	− 0.225	***
	CD2	− 0.186	***	− 0.208	***	− 0.210	***	− 0.226	***
B cell	CD19	− 0.275	***	− 0.306	***	− 0.257	***	− 0.307	***
	CD79A	− 0.240	***	− 0.261	***	− 0.191	***	− 0.232	***
Monocyte	CD86	0.021	0.629	0.042	0.353	− 0.138	**	− 0.147	**
	CSF1R	0.076	0.084	0.100	*	− 0.036	0.424	− 0.023	0.618
TAM	CCL2	0.047	0.288	0.064	0.155	− 0.014	0.756	− 0.014	0.759
	CD68	0.131	**	0.153	***	0.060	0.182	0.067	0.142
	IL10	− 0.054	0.224	− 0.044	0.330	− 0.085	0.058	− 0.097	*
M1 Macrophage	NOS2	− 0.032	0.464	− 0.032	0.482	− 0.131	**	− 0.133	**
	IRF5	0.005	0.910	0.007	0.882	− 0.012	0.792	− 0.003	0.944
	PTGS2	0.074	0.095	0.076	0.092	0.119	**	0.119	**
M2 Macrophage	CD163	0.075	0.090	0.100	*	− 0.025	0.575	− 0.013	0.779
	VSIG4	0.054	0.217	0.067	0.137	− 0.021	0.646	− 0.009	0.845
	MS4A4A	− 0.020	0.645	− 0.005	0.909	− 0.100	*	− 0.097	*
Neutrophils	CD66b	− 0.193	***	− 0.198	***	− 0.098	*	− 0.099	*
	CD11b	0.055	0.216	0.077	0.089	− 0.051	0.257	− 0.041	0.376
	CCR7	− 0.235	***	− 0.264	***	− 0.239	***	− 0.265	***
Natural killer cell	KIR2DL1	− 0.057	0.197	− 0.059	0.190	− 0.052	0.246	− 0.056	0.223
	KIR2DL3	0.073	0.098	0.079	0.080	− 0.141	**	− 0.127	**
	KIR2DL4	0.098	*	0.100	*	− 0.051	0.255	− 0.043	0.353
	KIR3DL1	− 0.023	0.605	− 0.030	0.504	− 0.072	0.106	− 0.061	0.180
	KIR3DL2	− 0.023	0.598	− 0.016	0.727	− 0.146	**	− 0.148	**
	KIR3DL3	0.061	0.165	0.058	0.195	0.013	0.769	0.003	0.948
	KIR2DS4	− 0.005	0.902	− 0.009	0.845	− 0.056	0.207	− 0.058	0.205
Dendritic cell	HLA-DPB1	− 0.166	***	− 0.167	***	− 0.131	**	− 0.140	**
	HLA-DQB1	− 0.111	*	− 0.107	*	− 0.100	*	− 0.100	**
	HLA-DRA	− 0.133	**	− 0.131	**	− 0.132	**	− 0.138	**
	HLA-DPA1	− 0.135	**	− 0.131	**	− 0.143	**	− 0.151	***
	ITGAX	− 0.016	0.710	− 0.009	0.837	− 0.119	**	− 0.135	**
	NRP1	0.013	0.768	0.005	0.904	0.071	0.110	0.082	0.0754
	CD1C	− 0.217	***	− 0.220	***	− 0.097	*	− 0.103	*
Th1	T-bet	− 0.125	**	− 0.137	**	− 0.240	***	− 0.258	***
	STAT4	− 0.112	*	− 0.121	**	− 0.159	***	− 0.177	***
	STAT1	0.109	*	0.122	**	− 0.049	0.276	− 0.056	0.225
	IFN-γ	− 0.021	0.637	− 0.015	0.735	− 0.149	**	− 0.155	**
	TNF-α	− 0.020	0.645	− 0.009	0.837	0.084	0.059	0.087	0.058
Th2	GATA3	0.012	0.792	0.031	0.494	0.011	0.809	0.015	0.742
	STAT6	− 0.050	0.256	− 0.055	0.222	− 0.038	0.394	− 0.042	0.359
	STAT5A	− 0.084	0.056	− 0.079	0.078	− 0.160	***	− 0.168	***
	IL13	− 0.092	*	− 0.086	0.057	− 0.133	**	− 0.146	**
Tfh	BCL6	− 0.057	0.196	− 0.061	0.178	− 0.106	*	− 0.116	*
	IL21	− 0.007	0.869	− 0.004	0.931	− 0.168	***	− 0.160	***
Th17	STAT3	− 0.067	0.129	− 0.066	0.142	0.112	*	0.114	*
	IL17A	− 0.083	0.059	− 0.093	*	− 0.074	0.099	− 0.074	0.105
Treg	FOXP3	− 0.035	0.424	− 0.032	0.477	− 0.124	**	− 0.127	**
	CCR8	− 0.084	0.057	− 0.080	0.078	− 0.125	**	− 0.132	**
	STAT5B	− 0.209	***	− 0.207	***	− 0.181	***	− 0.179	***
	TGFβ	0.061	0.166	0.069	0.128	0.235	***	0.241	***
T cell exhaustion	PD-1	− 0.036	0.420	− 0.035	0.436	− 0.237	***	− 0.256	***
	CTLA4	− 0.116	**	− 0.123	**	− 0.245	***	− 0.271	***
	LAG3	0.003	0.949	0.019	0.677	− 0.175	***	− 0.178	***
	TIM-3	0.041	0.355	0.061	0.177	− 0.111	*	− 0.113	*
	GZMB	0.075	0.087	0.090	*	− 0.184	***	− 0.196	***

*Cor* R value of Spearman’s correlation, *None* correlation without adjustment, *Purity* correlation adjusted by purity.

*p < 0.05, **p < 0.01, ***p < 0.001.

**Table 3:** Correlation analysis between *PKM2* and marker genes of immune cells in GEPIA.

Description	Gene markers	LUAD				LUSC			
		Normal		Tumor		Normal		Tumor	
		R	*p*	R	*p*	R	*p*	R	*p*
CD8+ T cell	CD8A	0.023	0.86	− 0.061	0.18	− 0.11	0.46	− 0.18	***
T cell (general)	CD3D	− 0.12	0.37	− 0.14	**	− 0.094	0.52	− 0.24	***
	CD3E	− 0.045	0.74	− 0.16	***	− 0.052	0.72	− 0.21	***
	CD2	− 0.11	0.41	− 0.13	**	− 0.073	0.61	− 0.22	***
B cell	CD19	0.021	0.88	− 0.27	***	0.054	0.71	− 0.16	***
	CD79A	0.035	0.79	− 0.28	***	− 0.1	0.48	− 0.19	***
Neutrophils	CD66b	0.029	0.83	− 0.11	*	0.13	0.37	− 0.1	*
	CCR7	0.081	0.54	− 0.18	***	0.0036	0.98	− 0.18	***
Dendritic cell	HLA-DPB1	0.17	0.2	− 0.12	**	0.35	*	− 0.13	**
	HLA-DQB1	− 0.044	0.74	− 0.11	*	− 0.076	0.6	− 0.083	0.067
	HLA-DRA	0.12	0.38	− 0.091	*	0.22	0.13	− 0.13	**
	HLA-DPA1	0.15	0.26	− 0.071	0.12	0.056	0.27	− 0.13	**
	CD1C	− 0.12	0.38	− 0.12	*	0.12	0.39	− 0.072	0.11
	ITGAX	0.35	**	− 0.037	0.42	0.3	*	− 0.089	*
Th1	T-bet	− 0.099	0.46	0.094	*	0.069	0.63	− 0.21	***
	STAT4	− 0.11	0.41	− 0.002	0.97	− 0.12	0.4	− 0.083	**
	STAT1	0.0099	0.94	0.26	***	0.36	**	0.029	0.53
	IFN-γ	− 0.17	0.2	0.057	0.21	− 0.26	0.07	− 0.12	**
Tfh	IL21	− 0.008	0.95	0.026	0.56	0.043	0.77	− 0.17	***
Th17	STAT3	0.14	0.29	0.11	*	0.3	*	0.16	***
Treg	FOXP3	0.15	0.25	− 0.021	0.64	0.077	0.6	− 0.12	**
	STAT5B	− 0.054	0.69	− 0.055	0.23	0.2	0.17	− 0.092	*
	TGFβ	0.46	***	0.17	***	0.53	***	0.33	***
T cell exhaustion	PD-1	0.082	0.54	0.011	0.82	0.069	0.63	− 0.22	***
	CTLA4	0.032	0.81	− 0.017	0.72	− 0.092	0.53	− 0.19	***
	LAG3	0.057	0.67	0.017	0.71	0.21	0.14	− 0.14	**
	GZMB	− 0.17	0.21	0.084	0.063	0.66	0.063	− 0.17	***

*p < 0.05, **p < 0.01, ***p < 0.001.

## Discussion

*PKM2* has been shown to be upregulated in various types of cancers and plays a crucial role in cancer metabolism^6^. Its functions extend beyond the regulation of glucose metabolic, as it has also been implicated in the modulation of intracellular reactive oxygen species levels and the maintenance of amino acid balance^14–16^. In addition, emerging evidence suggests that PKM2 could function as a protein kinase, mediating cancer progression, chemical resistance, and immunity regulation^17–19^.

Recent studies have demonstrated that *PKM2* is highly expressed in multiple cancers, and is closely associated with the disease progression, such as in pancreatic ductal adenocarcinoma (PDAC)^20^, colorectal cancer^21^, breast cancer^22^, and hepatocellular cancer^23^. In brain tumors, various findings demonstrated that in addition to its key role as a core regulator of cellular glycolysis, PKM2 holds a critical tumorigenic function as a protein kinase in the nucleus through its involvement in gene transcription and as a transcriptional co-activator of oncogenic signals^24^. Moreover, in bladder cancer (BCa), *PKM2* has been found to promote the growth, migration, and cisplatin resistance of BCa cells and may serve as a poor prognostic factor for BCa patients^25^. In our study, we found that *PKM2* mRNA expression levels were significantly upregulated in most human tumors using the GenT2, TIMER, and GEPIA databases. However, the expression of *PKM2* varied across different types of cancer, which could be attributed to variations in data collection methods and underlying pathogenic mechanisms^13^. Notably, tumor tissues consistently exhibited higher levels of *PKM2* expression compared to controls in lung cancer. High *PKM2* expression has been associated with poorer prognosis in various cancer types, including lung cancer, as revealed by survival analysis in the GEPIA and PrognoScan databases. Combining the findings from both databases, it became evident that high *PKM2* expression is detrimental to the prognosis of lung cancer patients.

As previously reported, *PKM2* expression could drive the metabolic reprogramming, promote proliferation, and induce glycolytic metabolism of NSCLC^26,27^. Additionally, *PKM2* has been implicated in promoting invasion and epithelial-mesenchymal transition (EMT) in lung cancer^28^. Our analysis, encompassing RNA-seq and gene chip data from both paired and unpaired cancer and normal tissues, consistently revealed higher PKM2 expression in lung cancer at both protein and mRNA levels. Furthermore, Kaplan–Meier Plotter analyses confirmed a significant correlation between high PKM2 expression and a worse prognosis in lung cancer, particularly in localized and regional early and mid-stage cancers. Collectively, these findings underscore the potential of PKM2 as a prognostic biomarker for lung cancer.

The composition and activity of infiltrating immune cells within the tumor microenvironment play a crucial role in shaping the immune response and have significant implications for the clinical prognosis of cancer patients^29^. Hou et al.^30^ and Li et al.^23^ have demonstrated that *PKM2* can drive hepatocellular carcinoma progression by inducing macrophage differentiation and inducing an immunosuppressive microenvironment. Our study demonstrated that *PKM2* expression was negatively correlated with B cells and CD4+ cells in LUAD and had a strong negative correlation with B cells, CD4+ and CD8+ T cells, and macrophages in LUSC. These findings suggest that *PKM2* plays a pivotal role in regulating tumor immunity in lung cancer, thereby influencing the prognosis of the patients. Previous studies have reported the presence of tumor-antagonizing immune cells within the tumor microenvironment, including CD8+ cells, natural killer (NK) cells, DCs, M1 macrophages, and neutrophils^31^. Our analysis revealed a significant negative correlation between *PKM2* expression and markers of CD8+ T cells, neutrophils, and DCs in both LUAD and LUSC. Moreover, in LUSC, *PKM2* was also negatively correlated with the expression of the M1 macrophage markers, NOS2 and PTGS2, and NK cell markers, KIR2DL3 and KIR3DL2. These results suggest that elevated *PKM2* expression may contribute to the progression of LUAD and LUSC by impeding the infiltration of tumor-antagonizing immune cells.

Previous studies have highlighted the nonmetabolic role of *PKM2* in modulating Th17 cell differentiation and function in autoimmune-mediated inflammation through enhanced STAT3 activation^17^. Our findings revealed a positive correlation between *PKM2* expression and STAT3, a marker for Th17 cells, in LUSC. Additionally, PKM2 expression also showed correlations with markers of various subsets of T helper (Th) cells, including Th1 (T-bet, STAT4, and IFN-γ), Th2 (STAT5A and IL13), Tfh (BCL6 and IL-21), and Tregs (FOXP3, CCR8, STAT5B, and TGF-β). In contrast, in LUAD, *PKM2* expression exhibited a significant negative correlation with markers of Th1 cells (T-bet, STAT4, and STAT1) and Treg cells (STAT5B). These results suggest a role for *PKM2* in regulating tumor-infiltration of T-helper cells. Interestingly, *PKM2* expression was also negatively correlated with markers of exhausted T cells especially in LUSC, where the expression of inhibitory immune checkpoint proteins PD-1, CTLA4, LAG3, and TIM-3^32^ were also negatively correlated with *PKM2* expression. Many cancers evade the immune response by overexpressing inhibitory ligands to suppress T-cell function, thereby promoting their own progression^33^. Accordingly, we speculated that this could explain the high expression of *PKM2* in LUSC tumor tissues despite its lack of close association with the prognosis of LUSC.

However, it is essential to acknowledge that our study has limitations due to its reliance on public resource databases. Nevertheless, the consistent findings across multiple databases substantiate the potential of *PKM2* as a prognostic biomarker for lung cancer and as an indicator of immune cell infiltration levels in LUAD and LUSC.

In conclusion, our study established a clear association between elevated *PKM2* expression and unfavorable prognosis in lung cancer patients, particularly those with localized and regional early and mid-stage lung cancers, by integrating data from multiple databases. More importantly, our study unveiled a correlation between *PKM2* and immune infiltration in lung cancer, providing novel insights for the treatment of lung cancer patients. Our study highlights the potential of *PKM2* as a promising therapeutic target in lung cancer.

## Materials and methods

### *PKM2* expression analysis

The Gent2 database (http://gent2.appex.kr/gent2/)^34^, TIMER (https://cistrome.shinyapps.io/timer/), and GEPIA (http://gepia.cancer-pku.cn/) were used to investigate the mRNA expression levels of the *PKM2* in human cancers^13,35^.

### TIMER database

TIMER is a comprehensive web server for systematic analysis of immune infiltration in various cancer types^36^. In this study, we used the “Diff Exp” module to study the expression difference of *PKM2* between tumors and adjacent normal tissues in diverse human cancers. The “Gene” module was used to explore the correlation of *PKM2* expression and the abundance of immune infiltrates, including B cells, CD8+ T cells, CD4+ T cells, macrophage, neutrophils and dendritic cells, in LUAD and LUSC. Furthermore, a correlation analysis was performed between *PKM2* expression and the expression of marker genes of infiltrating immune cells in LUAD and LUSC by the “Correlation” module^13^.

### GEPIA database analysis

The GEPIA database, which incorporates RNA sequencing data from 9736 tumor tissues and 8587 normal tissues sourced from TCGA and the Genotype-Tissue Expression (GTEx) databases, was utilized in this study^37^. Specifically, the “Boxplots” module of GEPIA was employed to analyze the expression of *PKM2* in various human cancers. The “Stage plot” module was used to investigate the relationship between *PKM2* expression and the pathological staging of LUAD and LUSC. Survival curves were generated using the “Survival Plots”, including OS and DFS, to assess the prognostic value of *PKM2*. The “correlation” module of the TIMER database was used to further validate the association between PKM2 expression and specific markers of distinct immune cell subsets^13^.

### Kaplan–Meier plotter

The data obtained from GEO, EGA and TCGA databases were analyzed using Kaplan Meier Plotter (http://kmplot.com/analysis/) to evaluate the correlation between the expression of all genes and the patient survival in more than 30,000 samples from 21 different tumor types^38^. To specifically investigate the role of *PKM2* expression in lung cancer, gene chip dataset was used to analyze the correlation between *PKM2* expression and prognosis as well as various clinicopathological factors^39^. The RNA-seq data was used to investigate the relationship between *PKM2* expression and the prognosis of LUAD and LUSC, including OS and RFS^40^. Meanwhile, the expression levels of *PKM2* in tumor tissues and adjacent normal tissues were also compared by using the Kaplan–Meier plotter database^41^.

### HPA

HPA (https://www.proteinatlas.org) database provides comprehensive proteomics, transcriptomics, and systems biology data^42^. The protein expression levels of PKM2 in both tumor tissues and adjacent normal tissues of LUAD and LUSC patients were investigated using the HPA database.

### PrognoScan database

PrognoScan (http://dna00.bio.kyutech.ac.jp/PrognoScan/PrognoScan.html) is a meta-analysis database for evaluating the prognostic value of genes^43^. The associations between *PKM2* expression and clinical outcomes in 12 types of cancers were determined with a *p* value < 0.05 as the threshold using PrognoScan^35^.

### Ethics statement

This study is based on summary data from public databases, which had gained written informed consent and ethics approval. No ethical approval is required for the secondary analysis of summary data.

### Supplementary Information

Supplementary Table S1.

Supplementary Table S2.

## Data Availability

The datasets generated during the current study are available from the corresponding author on reasonable request.
