# Supplementary material for: PKM2 is a potential prognostic biomarker and related to immune infiltration in lung cancer
Source: Sci Rep. 2023 Dec 14;13:22243. doi: 10.1038/s41598-023-49558-4 (PMC10721847; doi:10.1038/s41598-023-49558-4)
Supplement: Supplementary file 1 — Supplementary Table S1. [file 41598_2023_49558_MOESM1_ESM.docx]

| Cancer | OS | | |  | DFS | | |
| --- | --- | --- | --- | --- | --- | --- | --- |
|  | HR |  | Logrank p |  | HR |  | Logrank p |
| ACC | 1.8 |  | 0.13 |  | 1.9 |  | 0.053 |
| BLCA | 1.2 |  | 0.15 |  | 1 |  | 0.84 |
| BRCA | 1.2 |  | 0.19 |  | 0.95 |  | 0.78 |
| CESC | 1.8 |  | **0.015** |  | 1.1 |  | 0.74 |
| CHOL | 0.92 |  | 0.87 |  | 0.98 |  | 0.97 |
| COAD | 1.3 |  | 0.28 |  | 1.4 |  | 0.21 |
| DLBC | 0.92 |  | 0.91 |  | 1.1 |  | 0.89 |
| ESCA | 0.83 |  | 0.44 |  | 1 |  | 0.9 |
| GBM | 1.4 |  | 0.072 |  | 1.6 |  | **0.026** |
| HNSC | 1.3 |  | **0.035** |  | 1.1 |  | 0.7 |
| KICH | 3.7 |  | 0.081 |  | 1.7 |  | 0.42 |
| KIRC | 0.71 |  | **0.025** |  | 0.72 |  | 0.068 |
| KIRP | 1.2 |  | 0.56 |  | 1.3 |  | 0.33 |
| LAML | 1.9 |  | **0.026** |  | 1 |  | 1 |
| LGG | 1.3 |  | 0.11 |  | 0.94 |  | 0.7 |
| LIHC | 1.8 |  | **0.0015** |  | 1.3 |  | 0.1 |
| LUAD | 1.4 |  | **0.017** |  | 1.3 |  | 0.065 |
| LUSC | 1 |  | 0.82 |  | 1 |  | 0.79 |
| MESO | 2 |  | **0.0066** |  | 1.9 |  | **0.026** |
| OV | 1.2 |  | 0.13 |  | 0.86 |  | 0.22 |
| PAAD | 1.7 |  | **0.0097** |  | 1.6 |  | **0.046** |
| PCPG | 2 |  | 0.42 |  | 1.2 |  | 0.77 |
| PRAD | 1.9 |  | 0.34 |  | 1.2 |  | 0.31 |
| READ | 0.94 |  | 0.9 |  | 0.89 |  | 0.79 |
| SARC | 1.2 |  | 0.35 |  | 1.5 |  | **0.032** |
| SKCM | 1.3 |  | 0.055 |  | 1.2 |  | 0.24 |
| STAD | 0.99 |  | 0.93 |  | 0.98 |  | 0.92 |
| TCGT | 2.7 |  | 0.38 |  | 0.95 |  | 0.89 |
| THCA | 0.87 |  | 0.78 |  | 0.9 |  | 0.71 |
| THYM | 6.1 |  | 0.055 |  | 2.2 |  | 0.098 |
| UCEC | 1.4 |  | 0.4 |  | 1.1 |  | 0.71 |
| UCS | 1.2 |  | 0.51 |  | 1 |  | 0.98 |
| UVM | 4.3 |  | **0.0045** |  | 2.6 |  | **0.044** |

Table S1. Analysis of PKM2 expression in relation to OS and DFS of human cancer using the GEPIA database.

Bold values indicate P < 0.05.
