# Supplementary material for: PKM2 is a potential prognostic biomarker and related to immune infiltration in lung cancer
Source: Sci Rep. 2023 Dec 14;13:22243. doi: 10.1038/s41598-023-49558-4 (PMC10721847; doi:10.1038/s41598-023-49558-4)
Supplement: Supplementary file 2 — Supplementary Table S2. [file 41598_2023_49558_MOESM2_ESM.docx]

| **DATASET**  Table S2. Analysis of the prognostic value of PKM2 in human cancer using the PrognoScan database. | **CANCER TYPE** | **SUBTYPE** | **ENDPOINT** | **N** | **COX P-VALUE** | **HR [95% CIlow - CIupp]** |
| --- | --- | --- | --- | --- | --- | --- |
| GSE5287 | Bladder cancer |  | OS | 30 | 0.249462 | 1.36 [0.81 - 2.28] |
| GSE13507 | Bladder cancer |  | OS | 165 | 0.529174 | 0.90 [0.66 - 1.24] |
| GSE13507 | Bladder cancer | Transitional cell carcinoma | DSS | 165 | 0.479886 | 0.85 [0.53 - 1.35] |
| GSE12417-GPL96 | Blood cancer | AML | OS | 163 | 0.762219 | 0.97 [0.80 - 1.18] |
| GSE12417-GPL570 | Blood cancer | AML | OS | 79 | 0.179976 | 1.27 [0.90 - 1.81] |
| GSE5122 | Blood cancer | AML | OS | 58 | 0.173831 | 1.23 [0.91 - 1.67] |
| GSE8970 | Blood cancer | AML | OS | 34 | 0.670533 | 0.94 [0.71 - 1.25] |
| GSE4475 | Blood cancer | B-cell lymphoma | OS | 158 | 0.248164 | 0.76 [0.47 - 1.21] |
| E-TABM-346 | Blood cancer | DLBCL | DFS | 53 | 0.981904 | 1.01 [0.43 - 2.37] |
| E-TABM-346 | Blood cancer | DLBCL | OS | 53 | 0.65166 | 0.81 [0.32 - 2.04] |
| GSE16131-GPL96 | Blood cancer | Follicular lymphoma | OS | 180 | 0.557788 | 0.89 [0.60 - 1.32] |
| GSE2658 | Blood cancer | Multiple myeloma | DSS | 559 | **0.047669** | 1.47 [1.00 - 2.15] |
| GSE4271-GPL96 | Brain cancer | Astrocytoma | OS | 77 | 0.240823 | 1.29 [0.84 - 1.96] |
| GSE7696 | Brain cancer | Glioblastoma | OS | 70 | 0.761583 | 0.93 [0.58 - 1.48] |
| MGH-glioma | Brain cancer | Glioma | OS | 50 | **0.004323** | 2.27 [1.29 - 4.00] |
| MGH-glioma | Brain cancer | Glioma | OS | 50 | 0.284496 | 0.37 [0.06 - 2.28] |
| GSE4412-GPL96 | Brain cancer | Glioma | OS | 74 | **0.027091** | 2.60 [1.11 - 6.07] |
| GSE16581 | Brain cancer | Meningioma | OS | 67 | 0.198009 | 3.45 [0.52 - 22.67] |
| GSE19615 | Breast cancer |  | DMFS | 115 | 0.804381 | 1.20 [0.28 - 5.09] |
| GSE3143 | Breast cancer |  | OS | 158 | 0.481604 | 0.77 [0.37 - 1.59] |
| GSE3143 | Breast cancer |  | OS | 158 | 0.361155 | 0.87 [0.64 - 1.17] |
| GSE7849 | Breast cancer |  | DFS | 76 | 0.644709 | 0.80 [0.30 - 2.10] |
| GSE7849 | Breast cancer |  | DFS | 76 | 0.422359 | 0.79 [0.44 - 1.41] |
| GSE12276 | Breast cancer |  | RFS | 204 | 0.9229 | 1.01 [0.78 - 1.31] |
| GSE6532-GPL570 | Breast cancer |  | RFS | 87 | **0.035665** | 0.46 [0.23 - 0.95] |
| GSE6532-GPL570 | Breast cancer |  | DMFS | 87 | **0.035665** | 0.46 [0.23 - 0.95] |
| GSE9195 | Breast cancer |  | RFS | 77 | **0.005711** | 5.67 [1.66 - 19.39] |
| GSE9195 | Breast cancer |  | DMFS | 77 | **0.026109** | 4.58 [1.20 - 17.50] |
| GSE12093 | Breast cancer |  | DMFS | 136 | 0.347121 | 0.79 [0.49 - 1.28] |
| GSE11121 | Breast cancer |  | DMFS | 200 | 0.914072 | 1.05 [0.46 - 2.36] |
| GSE1378 | Breast cancer |  | RFS | 60 | 0.903344 | 1.06 [0.41 - 2.73] |
| GSE1379 | Breast cancer |  | RFS | 60 | 0.375003 | 1.40 [0.67 - 2.93] |
| GSE9893 | Breast cancer |  | OS | 155 | 0.105054 | 1.18 [0.97 - 1.44] |
| GSE2034 | Breast cancer |  | DMFS | 286 | 0.565065 | 0.90 [0.64 - 1.28] |
| GSE1456-GPL96 | Breast cancer |  | OS | 159 | 0.200562 | 1.47 [0.81 - 2.67] |
| GSE1456-GPL96 | Breast cancer |  | RFS | 159 | 0.31222 | 1.35 [0.75 - 2.43] |
| GSE1456-GPL96 | Breast cancer |  | DSS | 159 | 0.057889 | 1.96 [0.98 - 3.94] |
| GSE7378 | Breast cancer |  | DFS | 54 | 0.300109 | 1.62 [0.65 - 4.05] |
| GSE7378 | Breast cancer |  | DFS | 54 | 0.59403 | 0.82 [0.40 - 1.70] |
| GSE7378 | Breast cancer |  | DFS | 54 | 0.315956 | 0.12 [0.00 - 7.41] |
| E-TABM-158 | Breast cancer |  | DMFS | 117 | 0.152948 | 0.61 [0.31 - 1.20] |
| E-TABM-158 | Breast cancer |  | RFS | 117 | 0.957425 | 1.02 [0.58 - 1.78] |
| E-TABM-158 | Breast cancer |  | OS | 117 | 0.957425 | 1.02 [0.58 - 1.78] |
| E-TABM-158 | Breast cancer |  | DSS | 117 | 0.718002 | 0.89 [0.46 - 1.72] |
| GSE3494-GPL96 | Breast cancer |  | DSS | 236 | **0.026267** | 2.41 [1.11 - 5.24] |
| GSE4922-GPL96 | Breast cancer |  | DFS | 249 | **0.023162** | 2.09 [1.11 - 3.96] |
| GSE2990 | Breast cancer |  | DMFS | 125 | 0.27296 | 1.36 [0.78 - 2.36] |
| GSE2990 | Breast cancer |  | DMFS | 54 | 0.555011 | 0.85 [0.50 - 1.46] |
| GSE2990 | Breast cancer |  | RFS | 125 | 0.433865 | 1.19 [0.77 - 1.82] |
| GSE2990 | Breast cancer |  | RFS | 62 | 0.510216 | 0.86 [0.55 - 1.34] |
| GSE7390 | Breast cancer |  | RFS | 198 | 0.450126 | 1.12 [0.83 - 1.52] |
| GSE7390 | Breast cancer |  | DMFS | 198 | 0.117299 | 1.34 [0.93 - 1.94] |
| GSE7390 | Breast cancer |  | OS | 198 | 0.262038 | 1.25 [0.85 - 1.83] |
| GSE12945 | Colorectal cancer |  | DFS | 51 | 0.522984 | 0.45 [0.04 - 5.30] |
| GSE12945 | Colorectal cancer |  | OS | 62 | **0.012029** | 2.87 [1.26 - 6.52] |
| GSE17536 | Colorectal cancer |  | DFS | 145 | **0.015122** | 2.22 [1.17 - 4.22] |
| GSE17536 | Colorectal cancer |  | OS | 177 | **0.007398** | 1.90 [1.19 - 3.03] |
| GSE17536 | Colorectal cancer |  | DSS | 177 | **0.005065** | 2.17 [1.26 - 3.72] |
| GSE14333 | Colorectal cancer |  | DFS | 226 | **0.028539** | 1.71 [1.06 - 2.76] |
| GSE17537 | Colorectal cancer |  | OS | 55 | 0.701302 | 1.14 [0.58 - 2.28] |
| GSE17537 | Colorectal cancer |  | DFS | 55 | 0.746585 | 1.14 [0.52 - 2.49] |
| GSE17537 | Colorectal cancer |  | DSS | 49 | 0.933521 | 1.04 [0.41 - 2.64] |
| GSE22138 | Eye cancer | Uveal melanoma | DMFS | 63 | **0.042321** | 1.49 [1.01 - 2.20] |
| GSE2837 | Head and neck cancer | Squamous cell carcinoma | RFS | 28 | 0.19339 | 1.25 [0.89 - 1.75] |
| GSE2837 | Head and neck cancer | Squamous cell carcinoma | RFS | 28 | 0.199515 | 1.32 [0.86 - 2.01] |
| jacob-00182-CANDF | Lung cancer | Adenocarcinoma | OS | 82 | 0.075248 | 2.24 [0.92 - 5.44] |
| HARVARD-LC | Lung cancer | Adenocarcinoma | OS | 84 | **0.045437** | 2.15 [1.02 - 4.55] |
| jacob-00182-HLM | Lung cancer | Adenocarcinoma | OS | 79 | 0.66729 | 1.18 [0.55 - 2.51] |
| MICHIGAN-LC | Lung cancer | Adenocarcinoma | OS | 86 | 0.161658 | 2.42 [0.70 - 8.38] |
| jacob-00182-MSK | Lung cancer | Adenocarcinoma | OS | 104 | 0.008427 | 4.16 [1.44 - 12.04] |
| GSE13213 | Lung cancer | Adenocarcinoma | OS | 117 | 0.226226 | 1.41 [0.81 - 2.47] |
| GSE13213 | Lung cancer | Adenocarcinoma | OS | 117 | 0.566283 | 1.17 [0.69 - 1.99] |
| GSE31210 | Lung cancer | Adenocarcinoma | OS | 204 | **0.000001** | 4.56 [2.47 - 8.44] |
| GSE31210 | Lung cancer | Adenocarcinoma | RFS | 204 | **0** | 3.50 [2.24 - 5.45] |
| jacob-00182-UM | Lung cancer | Adenocarcinoma | OS | 178 | 0.37005 | 1.34 [0.70 - 2.56] |
| GSE11117 | Lung cancer | NSCLC | OS | 41 | 0.840068 | 1.07 [0.57 - 1.98] |
| GSE11117 | Lung cancer | NSCLC | OS | 41 | 0.635083 | 0.86 [0.47 - 1.59] |
| GSE3141 | Lung cancer | NSCLC | OS | 111 | **0.021685** | 1.64 [1.07 - 2.50] |
| GSE14814 | Lung cancer | NSCLC | OS | 90 | 0.949803 | 1.02 [0.59 - 1.75] |
| GSE14814 | Lung cancer | NSCLC | DSS | 90 | 0.689282 | 0.88 [0.49 - 1.61] |
| GSE8894 | Lung cancer | NSCLC | RFS | 138 | 0.086436 | 1.25 [0.97 - 1.61] |
| GSE4573 | Lung cancer | Squamous cell carcinoma | OS | 129 | **0.018269** | 3.04 [1.21 - 7.67] |
| GSE17710 | Lung cancer | Squamous cell carcinoma | RFS | 56 | 0.641972 | 1.19 [0.58 - 2.44] |
| GSE17710 | Lung cancer | Squamous cell carcinoma | OS | 56 | 0.506831 | 1.28 [0.62 - 2.64] |
| GSE17710 | Lung cancer | Squamous cell carcinoma | RFS | 56 | 0.70594 | 1.12 [0.62 - 2.03] |
| GSE17710 | Lung cancer | Squamous cell carcinoma | OS | 56 | 0.569928 | 1.19 [0.66 - 2.13] |
| GSE17710 | Lung cancer | Squamous cell carcinoma | RFS | 56 | 0.805792 | 1.09 [0.55 - 2.15] |
| GSE17710 | Lung cancer | Squamous cell carcinoma | OS | 56 | 0.618689 | 1.19 [0.61 - 2.32] |
| GSE17710 | Lung cancer | Squamous cell carcinoma | RFS | 56 | 0.606108 | 1.18 [0.62 - 2.24] |
| GSE17710 | Lung cancer | Squamous cell carcinoma | OS | 56 | 0.44468 | 1.28 [0.68 - 2.40] |
| GSE9891 | Ovarian cancer |  | OS | 278 | 0.579265 | 0.92 [0.68 - 1.24] |
| DUKE-OC | Ovarian cancer |  | OS | 133 | 0.933941 | 1.01 [0.75 - 1.38] |
| GSE8841 | Ovarian cancer |  | OS | 81 | 1 | 1.00 [1.00 - 1.00] |
| GSE8841 | Ovarian cancer |  | OS | 81 | 0.809863 | 0.93 [0.53 - 1.63] |
| GSE26712 | Ovarian cancer |  | OS | 185 | 0.519329 | 0.95 [0.80 - 1.12] |
| GSE26712 | Ovarian cancer |  | DFS | 185 | 0.460723 | 0.94 [0.81 - 1.10] |
| GSE17260 | Ovarian cancer |  | PFS | 110 | 0.601157 | 0.91 [0.64 - 1.29] |
| GSE17260 | Ovarian cancer |  | OS | 110 | 0.766995 | 0.94 [0.61 - 1.45] |
| GSE17260 | Ovarian cancer |  | PFS | 110 | 0.494431 | 0.87 [0.59 - 1.29] |
| GSE17260 | Ovarian cancer |  | OS | 110 | 0.844123 | 0.95 [0.59 - 1.55] |
| GSE14764 | Ovarian cancer |  | OS | 80 | 0.988567 | 1.00 [0.56 - 1.78] |
| GSE16560 | Prostate cancer |  | OS | 281 | **0.000154** | 2.66 [1.60 - 4.41] |
| GSE19234 | Skin cancer | Melanoma | OS | 38 | 0.090946 | 2.57 [0.86 - 7.66] |
| GSE30929 | Soft tissue cancer | Liposarcoma | DRFS | 140 | **0.000027** | 1.95 [1.43 - 2.66] |

Bold values indicate P < 0.05
